# Mental Health Status of the General Public, Frontline, and Non-frontline Healthcare Providers in the Early Stage of COVID-19

**DOI:** 10.3389/fpsyt.2021.553021

**Published:** 2021-03-18

**Authors:** Dan Luo, Qian Liu, Qin Chen, Run Huang, Pan Chen, Bing Xiang Yang, Zhongchun Liu

**Affiliations:** ^1^Department of Nursing, School of Health Sciences, Wuhan University, Wuhan, China; ^2^Department of Psychiatry, Renmin Hospital of Wuhan University, Wuhan, China; ^3^Neurosurgery Department, Zhongnan Hospital of Wuhan University, Wuhan, China

**Keywords:** COVID-19, healthcare providers, general public, mental health distress, depression, anexity

## Abstract

**Background:** The outbreak of COVID-19 occurred in 2020 which resulted in high levels of psychological stress in both the general public and healthcare providers.

**Purpose:** The study aimed to address the mental health status of people in China in the early stage of the COVID-19 outbreak, and to identify differences among the general public, frontline, and non-frontline healthcare providers.

**Method:** A cross-sectional study was used to identify the mental health status of the general public and healthcare providers between Jan 29 and Feb 11, 2020. Data were collected using an online survey from a convenience sample. The instruments used included: Patient Health Questionnaire, Generalized Anxiety Disorder scale, Insomnia Severity Index, and Impact of Event Scale-Revised. Descriptive statistics were used to describe the data. Kruskal-Wallis H tests were performed to assess differences in measurements among the three groups; *P* < 0.05 (two-sided) was considered to be statistically significant.

**Results:** Results showed that a majority of participants experienced post-traumatic stress (68.8%), depression (46.1%), anxiety (39.8%), and insomnia (31.4%). Significant changes in the mental health status of frontline providers was found as compared to those of the other groups (*P* < 0.001). Interestingly, the scores of the general public were significantly higher than those of the non-frontline healthcare providers (*P* < 0.001).

**Conclusion:** These findings provide information to evaluate outbreak associated psychological stress for the general public and healthcare providers, and assist in providing professional support and actionable guidance to ease psychological stress and improve mental health.

## Introduction

The outbreak of Coronavirus Disease (COVID-19) occurred in Hubei province in December 2019, resulting in more than 82,165 confirmed cases, and 3,298 deaths in China (1). The pandemic then spread quickly world-wide as countries rolled out measures to curb the effects of the novel coronavirus (2). In the face of this large-scale public health event, both healthcare providers and the public have been experiencing psychological pressure. The surge of confirmed cases and deaths stressed the entire healthcare system, and many healthcare providers were recruited from multiple departments to control the epidemic. About nine million residents of Wuhan (the provincial capital) were under home quarantine for 2 months because of the lock-down policy, and their life was significantly disrupted (3). The development and implementation of mental health assessment, treatment and services are vital goal in the health response to the COVID-19 outbreak (4).

Following the major outbreaks of severe respiratory syndrome (SARS) in 2003 and Middle East respiratory syndrome (MERS) in 2015, a series of mental health disorders of healthcare workers were reported, including depression, anxiety, delirium, post-traumatic stress disorder (PTSD), and even suicidality (5, 6). The risk factors for these mental health disorders included exposure to trauma, such as witnessing and caring for patients who were severely ill, the deaths of healthcare professionals, substantial mortality and bereavement, perceived life threat, orphaning of children, food and resource insecurity, discrimination against affected families, and stigma (7). Although the outbreaks of SARS and MERS stimulated related research, there has been minimal focus on the psychological impact of infectious diseases on persons in the initial stage of the outbreak.

This study aims to understand the changes in the mental health status of the general public and healthcare providers in the early stage of the COVID-19 outbreak. The findings of this study will provide insight into the development of psychological interventions aimed to support people affected by a pandemic.

## Methods

### Settings and Participants

Due to the strict lockdown and quarantine policy, this national, cross-sectional study was conducted between Jan 20 and February 11, 2020 using an online survey. Ethical approval for this study was received from the institutional review board at Renmin Hospital of Wuhan University (No. WDRY2020-K004). Eligibility criteria of participants included: (1) frontline healthcare providers: a licensed healthcare professional who worked in a hospital designated to care for COVID-19 patients; (2) non-frontline healthcare providers: a licensed health professional who worked in a health care facility that did not directly care for COVID-19 patients; (3) members of the general public: residents of the community who were >18 years of age. Staff from the COVID-19 designated hospitals were contacted by the research team and asked to invite members of their work group to complete an online survey. This survey was distributed *via* WeChat, a commonly used social media platform in China. Meanwhile, information about the study and the online survey link were posted on WeChat to recruit participants from the general public. In this way, the online survey was open to a large population of healthcare providers and the general public. Respondents were asked to complete an online informed consent, prior to completing the survey. A total of 915 frontline healthcare providers, 1,659 non-frontline healthcare providers and 490 members of the general public were recruited and completed the survey.

### Demographic and Mental Health Questionnaires

Demographic questionnaires collected data on gender, age, marital status, educational background, profession, and professional titles.

#### Depression

Depression was evaluated by the Patient Health Questionnaire (PHQ-9), which has nine items measuring self-assessed depressive symptoms experienced during the previous 2 weeks. It uses a 4-point Likert-type scale (0 = never, 1 = sometimes, 2 = more than once a week, and 3 = almost every day). The total score ranges from zero to 27, and higher scores indicate more depressive symptoms. Scores of 5, 10, and 15 represent cutpoints for mild, moderate, and moderately severe depression, respectively. The PHQ-9 has shown good psychometric properties (8, 9).

#### Anxiety

Anxiety was measured by the Generalized Anxiety Disorder scale (GAD-7), a self-report tool developed by Spitzer et al. (10) that follows the criteria from the Diagnostic and Statistical Manual-IV (DSM-IV). The seven items included continuous variables and verification questions. These items describe the typical symptoms of generalized anxiety disorder (GAD) and are rated on a 4-point scale, from “not at all” to “nearly every day”. The scores on the nine items are summed for total scores that range from zero to 27; a higher score represents higher anxiety severity. Scores of 5, 10, and 15 represent cutpoints for mild, moderate, and moderately severe anxiety, respectively. Psychometric evaluations of the GAD-7 suggest that it is a reliable and valid measure of GAD symptoms in the psychiatric patient (11, 12).

#### Insomnia

The Insomnia Severity Index (ISI) is a brief instrument that assesses insomnia according to the criteria from the DSM-IV and the International Classification of Sleep Disorders (13). The ISI is a 7-item self-report questionnaire assessing the nature, severity, and impact of insomnia in the past month. A 5-point Likert scale (0 = none; 4 = very severe) is used to rate each item, with total scores ranging from zero to 28. A higher total score indicates more severe sleep difficulties. Scores of 8, 15, and 22 represent cutpoints for subthreshold, moderate, and moderately severe insomnia, respectively. Adequate psychometric properties for both the English and Chinese versions have been reported in previous studies (14, 15).

#### Post-traumatic Stress

The Impact of Event Scale (IES), a self-report questionnaire, is the most widely used measure of PTSD symptoms in critical care outcomes research (16). The IES-R which is the revised version of the scale measures reexperiencing (intrusion) symptoms, avoidance/numbing symptoms, and hyperarousal symptoms of PTSD (17). With the IES-R, respondents are asked to report how distressed or bothered they have been by particular difficulties in the past seven days: “not at all” (item score 0), “a little bit” (score, 1), “moderately” (score, 2), “quite a bit” (score, 3), or “extremely” (score, 4), with total scores ranging from zero to 88 for the 22 items of the scale. Scores of 9, 26, and 44 represent cutpoints for mild, moderate, and moderately severe PTSD symptoms, respectively. The IES-R has good reliability and validity in both the English and Chinese versions (18–20).

### Statistical Analysis

Descriptive statistics including frequency and percentages were used to describe the demographic characteristics of the participants. Since the data were not fit the normal distribution, Kruskal-Walls H tests were performed to assess differences in the characteristics and severity of mental health distress between frontline, non-frontline healthcare providers and the general public and *P* < 0.05 (two-sided) was considered to be statistically significant. Furthermore, Kruskal-Walls H tests were performed to assess the differences in the total score of the mental health measurements between the three groups of participants. Dunn-Bonferroni *post-hoc* tests were utilized to compare the group differences when the result of the Kruskal-Walls H tests indicated a statistical significance.

## Results

### Comparison of Demographic Characteristics Between the General Public, Non-frontline, and Frontline Healthcare Providers

A total of 3,064 participants (915 frontline healthcare providers, 1,659 non-frontline healthcare providers, and 490 members of the general public) completed the online survey. Most were female (75.8%) and the majority (76.1%) were <40 years of age. Significant statistical differences were found in gender, age, marital status, and educational background between the general public, non-frontline, and frontline healthcare providers. Additionally, there was a significant difference in professional titles between non-frontline and frontline healthcare providers (see Table 1).

**Table 1:** Socio-demographic characteristics of participants (general public, frontline, and non-frontline) (*N* = 3,064).

**Variables**	**Number (%)**				**X^**2**^**	***P*-value**
	**Total (*N* = 3064)**	**General public (*n* = 490)**	**Non-frontline (*n* = 1659)**	**Frontline (*n* = 915)**		
**Gender**						
Male	741 (24.2)	203 (41.1)	361 (21.8)	177 (19.3)		-
Female	2323 (75.8)	287 (58.6)	1298 (78.2)	738 (80.7)		
**Age (in years)**						
18~30	1351 (44.1)	189 (38.6)	697 (42.1)	465 (50.8)		-
31~40	980 (32.0)	172 (35.1)	513 (30.9)	295 (32.2)		
>41	733 (23.9)	129 (26.3)	449 (27.1)	155 (16.9)		
**Marital status**						
Single	1047 (34.2)	171 (34.9)	499 (30.1)	377 (41.2)		-
Married	2017 (65.8)	319 (65.1)	1160 (69.9)	538 (58.8)		
**Educational background**						
Bachelor's degree and below	2551 (83.3)	425 (86.7)	1361 (82.0)	765 (83.6)		-
Master's degree and above	513 (16.7)	65 (13.3)	298 (18.0)	150 (16.4)		
**Profession**						
Physician	NA	NA	535 (32.2)	248 (27.1)		-
Nurse			947 (57.1)	640 (69.9)		
Other			177 (10.7)	27 (3.0)		
**Professional title**						
Junior	NA	NA	910 (54.9)	566 (61.8)		-
Intermediate			457 (27.5)	254 (27.8)		
Senior			292 (17.6)	95 (10.4)		
**PHQ-9**						
None	1651 (53.9)	264 (53.9)	1010 (60.9)	377 (41.2)	95.021	<0.001
Mild	933 (30.5)	127 (25.9)	458 (27.6)	348 (38.0)		
Moderate	277 (9.0)	52 (10.6)	111 (6.7)	114 (12.5)		
Moderately severe	203 (6.6)	47 (9.6)	80 (4.8)	76 (8.3)		
**GAD-7**						
None	1843 (60.2)	301 (61.4)	1108 (66.8)	434 (47.4)	95.174	<0.001
Mild	865 (28.2)	125 (25.5)	409 (24.7)	331 (36.2)		
Moderate	218 (7.1)	42 (8.6)	84 (5.1)	92 (10.1)		
Moderately severe	138 (4.5)	22 (4.5)	58 (3.5)	58 (6.3)		
**ISI**						
None	2103 (68.6)	348 (71.0)	1226 (73.9)	529 (57.8)	81.630	<0.001
Subthreshold	554 (23.9)	111 (22.7)	348 (21.0)	268 (29.3)		
Moderate	167 (7.2)	28 (5.7)	75 (4.5)	104 (11.4)		
Moderately severe	19 (0.8)	3 (0.6)	10 (0.6)	14 (1.5)		
**IES-R**						
None	955 (31.2)	153 (31.2)	583 (35.1)	219 (23.9)	87.197	<0.001
Mild	1121 (36.6)	181 (36.9)	647 (39.0)	293 (32.0)		
Moderate	703 (22.9)	110 (22.4)	325 (19.6)	268 (29.3)		
Moderately severe	285 (9.3)	46 (9.4)	104 (6.3)	135 (14.8)		

### Comparison of Depression, Anxiety, Insomnia, and Impact of Event Scores Between the General Public, Non-frontline, and Frontline Healthcare Providers

In this study, a majority (68.8%) of participants experienced post-traumatic stress and some reported symptoms of depression (46.1%), anxiety (39.8%), and insomnia (31.4%). When comparing participants in the three groups, more frontline healthcare workers had depression (58.8%), anxiety (52.6%), insomnia (42.2%), and post-traumatic stress (76.1%) than participants in the other groups. The results showed significant differences in the occurrence of depression, anxiety, insomnia, and post-traumatic stress in these groups (*P* < 0.001) (see Table 1).

Results in Table 2 show that a statistically significant difference was found among the three groups of participants (general public, non-frontline health professionals, and frontline health professionals) in regard to four aspects of mental health status. The frontline healthcare providers had significantly higher median scores for depression, anxiety, insomnia, and post-traumatic stress than the general public and non-frontline healthcare providers (*P* < 0.001). The general public had significantly higher median scores for depression, anxiety, and insomnia than non-frontline healthcare providers (*P* < 0.001).

**Table 2:** Characteristics of mental health status of participants (general public, frontline, and non-frontline) (*N* = 3,064).

	**N**	**Median (Interquartile range)**	***X^**2**^***	***P*-Value**
**PHQ-9**				
General Public	490	4 (1–9)^*^1^**^3	91.897	<0.001
Non-frontline	1659	3 (1–7)^*^1^**^2		
Frontline	915	6 (2–9)^**^2^**^3		
**GAD-7**				
General Public	490	3 (0–7)^**^1^**^3	91.874	<0.001
Non-frontline	1659	2 (0–6)^**^1^**^2		
Frontline	915	5 (1–8)^**^2^**^3		
**ISI**				
General Public	490	4 (1–9)^*^1^**^3	61.658	<0.001
Non-frontline	1659	3 (1–8)^*^1^**^2		
Frontline	915	6 (2–11)^**^2^**^3		
**IES-R**				
General Public	490	17 (7–30)^**^2	68.534	<0.001
Non-frontline	1659	15 (5–26)^**^1		
Frontline	915	23 (9–36)^**^1^**^2		

* P < 0.05;

** *P < 0.01; number 1, 2, and 3 are paired groups which were compared using Dunn-Bonferroni post-hoc tests*.

## Discussion

Most of the participants were under 40 years of age. This might be due to the fact that the social media platform used to distribute the survey is more often accessed by young adults. The results indicated that the frontline healthcare team is younger (<age 41), and many were single, with junior-level professional titles. This is consistent with the fact that hospitals are the traditional location of employment of recent healthcare graduates. More nurses (69.9%) were in the frontline team in Hubei province and this is consistent with nurses comprising 68% of the frontline team throughout China due to the intensive care needs of patients with COVID-19. Therefore, the sample in this study was representative of the healthcare providers.

The results of this study indicated that during the initial stage of the COVID-19 outbreak, healthcare providers who provided direct care for patients with COVID-19 had significantly higher scores on depression, anxiety, insomnia, and post-traumatic stress. This result was consistent with the findings of previous research during the outbreaks of SARS and MERS, as most of the healthcare providers experienced severe emotional distress (21–23). When the coronavirus appeared, frontline providers were managing this unknown infectious disease and adapting their expertise to stop its rapid spread. They were faced with a myriad of physical and psychological challenges, including intensive work for long hours, an entirely new disease, shortages of equipment and supplies, high risk of occupational exposure, fear of spreading the virus to their families or colleagues, caring for patients who were critically ill, and witnessing the sudden loss of lives (4). All these physical and psychological stressors make frontline healthcare providers particularly vulnerable to mental health distress. To curb this global public health crisis, healthcare providers are the most valuable resources for every country. It is crucial to assess their mental health status regularly, and provide comprehensive support to protect their well-being, including establishing a safe working environment and reasonable work schedules, providing sufficient personal protective equipment and continuous monitoring, and supervision of infection prevention strategies. Professional psychological counseling and crisis management interventions should be made available when necessary.

This study also showed that the outbreak of COVID-19 impacted everyone. At the end of January, most of the provinces and municipalities launched level I emergency responses, the highest level for a public health emergency. In Hubei province, the epicenter of the outbreak, cities were on lockdown and public transport was suspended. Residents were required to conduct self-quarantine at home to contain the spread of COVID-19. They had more time to view information about the epidemic on television and online social media. The increased availability of information made the general public more aware of the situation, but it also added to their fear and anxiety. The surge in confirmed cases and deaths, the deaths of infected healthcare personnel, as well as the need for individuals to monitor their temperature and maintain strict quarantine policies, raised the public's awareness but also sent out alarm signals. Indirect exposure to extreme events through repeated presentation and overloaded information from the news media, might create distress and elevate risks for common mental health disorders (24). Meanwhile, myths and misinformation were often driven by news reports and inaccurate public health messaging. For example, rumors circulated that eating fish would increase the risk of infection because they feed on waste products of poultry and livestock which could be infected; the virus could be transmitted by a mosquito bite, etc. The overwhelming media reporting and not knowing how to discern what information is relevant can lead to panic, fear, and anxiety.

The quarantine requirement for the general public was another major issue to consider. Individuals lacked diversity in their support from others, social interactions to validate their personal perspectives and a means of expressing their concerns. They may experience boredom, loneliness and anger (4). This can increase the risk for a high prevalence rate for symptoms of psychological distress (e.g., depression, stress, insomnia, irritability, and post-traumatic stress) (25). To decrease the public's sense of uncertainty and fear in a public health crisis, government officials and the media need to provide timely, accurate and transparent information about the epidemic, emphasize the importance of self-quarantine, explain how long it will continue, provide meaningful indoor activities and practical advice on coping and stress management, ensure the availability of basic supplies, and suggest professional support when necessary (4, 25).

The non-frontline healthcare providers also experienced psychological stressors, due to the severe shortage of personal protective equipment, which was mainly supplied to the frontline. They were worried about inadequate protection and increased risk of infection, because their patients might be in an incubation period without manifesting any symptoms of COVID-19, or hide their history of exposure to confirmed, suspected cases or epidemic areas (26). A study in Wuhan found that out of 40 infected healthcare workers, 31 (77.5%) worked on general wards (27). To prevent cross-contamination in hospitals, non-frontline departments were temporarily closed. Therefore, the workload of non-frontline healthcare providers was decreased compared with that of the frontline teams. The results also indicated that non-frontline healthcare workers had less mental health distress compared with the general public group. This may be due to the educational background of the non-frontline health providers who were able to distinguish facts from rumors compared with the general public. As a result, the providers were more likely to adapt in the midst of this public health crisis.

## Strengths and Limitations

This study described and compared the mental health status of the general public, non-frontline, and frontline healthcare in China in the early stage of the COVID-19 outbreak. It provided a wealth of information and gain a representative picture of the psychological response from a large group of population in this stress-coping period.

There are two major limitations in this study that could be addressed in future research. First, a cross-sectional study does not explore the causal relationship. Second, sampling bias is a concern when using convenience sampling methods. The sample recruited may be systematically different from the general population, which may cause the study to be biased and limit the generalizability of the results. But gender distribution of general public participants was nearly even between males and females, and the majority of healthcare providers who responded were nurses, which in China, is almost exclusively a female occupation. Thus, the participants were still representative in this study regarding the general public and healthcare provider groups. Therefore, a longitudinal large-scale study which enrolls more male healthcare providers is necessary to explore the impact of an infectious disease outbreak and to explore possible predictors of changes in mental health status.

## Conclusion

The general public, frontline, and non-frontline healthcare providers experienced different changes in their mental health status. The frontline healthcare providers reported more manifestations of depression, anxiety, insomnia, and post-traumatic stress. Effective prevention and response measures are essential to address the mental health issues associated with population-wide exposure during the early phase of the COVID-19 crisis. It is necessary to assess for early outbreak associated psychological stressors in both the general public and healthcare providers, and provide professional support and actionable guidance to ease their pressures and improve mental health.

## Data Availability Statement

The raw data supporting the conclusions of this article will be made available by the authors, without undue reservation.

## Ethics Statement

The studies involving human participants were reviewed and approved by the institutional review board at Renmin Hospital of Wuhan University (No. WDRY2020-K004). The patients/participants provided their written informed consent to participate in this study.

## Author Contributions

BY and ZL have full access to all the data in the study, take responsibility for the integrity of the data, the accuracy of the data analysis, and supervision. BY, DL, QL, and QC: conceptualization, methodology, and data curation. QL and DL: writing-original draft preparation. QC, RH, and DL: software and validation. QL, DL, PC, and BY: writing-reviewing and editing. All authors contributed to the article and approved the submitted version.

## Conflict of Interest

The authors declare that the research was conducted in the absence of any commercial or financial relationships that could be construed as a potential conflict of interest.

## Abbreviations

PHQ-9: patient health questionnaire
GAD-7: generalized anxiety disorder
ISI: insomnia severity index
ISE-R: impact of event scale-revised.
